# Educational Interventions for Children and Youth with Autism: A 40-Year Perspective

**DOI:** 10.1007/s10803-021-04990-1

**Published:** 2021-04-18

**Authors:** Samuel L. Odom, Laura J. Hall, Kristi L. Morin, Bonnie R. Kraemer, Kara A. Hume, Nancy S. McIntyre, Sallie W. Nowell, Jessica R. Steinbrenner, Brianne Tomaszewski, Ann M. Sam, Leann DaWalt

**Affiliations:** 1 University of North Carolina At Chapel Hill, Chapel Hill, NC USA; 2 San Diego State University, San Diego, CA USA; 3 Lehigh University, Bethlehem, PA USA; 4 University of Central Florida, Orlando, FL USA; 5 University of Wisconsin, Madison, WI USA; 6 Frank Porter Graham Child Development Institute, University of North Carolina at Chapel Hill, CB 8040, Chapel Hill, NC 27599-8040 USA

**Keywords:** Educational Interventions, Schools

## Abstract

Commemorating the 40 th anniversary of the Diagnostic and Statistical Manual (DSM) III, the purpose of this commentary is to describe school-based and school-relevant interventions and instructional approaches for children and youth with autism that have been developed and employed during that time period. The commentary begins with a brief description of foundational research that provides an historical context. Research themes shaped by science, ethics, social policy, and the changes in the DSM provide an organization for describing the evolution of intervention and instructional practices over the four previous decades. The commentary concludes with a discussion of school-contextual variables that influence implementation and the promise of the “iSciences” for closing the research to practice gap in the future.

In 1980 the American Psychiatric Association was finalizing diagnostic criteria that would shape the face of autism^1^ in the future, and public schools in the United States (U.S.) had been offering educational services to children and youth with disabilities, as mandated by a federal law, for about four years. At that time, autism was a very low-prevalence disorder, occurring in only 2–5 children per 10,000 (National Research Council, 2001). Autism had not been established as an eligibility category for receiving special education services, although autistic children and youth were enrolled in special education under other eligibility definitions (e.g., mental retardation, other health impaired, severe emotional disorder). Across these years, the intervention landscape in education has changed drastically. In 2018, public schools in the United States provided special education services to 663,098 school-aged children and youth with autism (U. S. Department of Education, 2021), as compared to 18,903 in 1993 (U. S. Department of Education, 1995). As the context has changed over the years, important intervention practices have evolved– shaped by science, ethics, policy trends, and the changes in the Diagnostic and Statistical Manual (DSM). The purpose of this commentary is to examine this evolution, highlighting practices and interventions that have become, or could become, the technology and tradecraft of education as it is provided by U.S. educational systems. Although the educational system in the United States is the focus of this review, we propose that the evolution and identification of current practices has implications for the broader international context.

In the initial section of the paper we briefly describe the historical scientific precedents to practices that emerged in the intervention literature. The second section of this commentary will identify practices organized within themes and influences in education and intervention research over the last 40 years. The commentary will conclude with discussion of the research to practice gap and factors in the context of public schools that affect adoption and implementation.

## Parameters of the Commentary

This paper is a commentary and not a systematic review. As such, it represents the perspectives of an informed research group that has decades of experience conducting and publishing research on school-based interventions for autistic children and youth. The grade range and approximate ages of participants extends from preschool, which begins at age 3, thorough graduation and post-school transition programs, which can be up to age 21. Discussion of early intervention for autistic children below the age of 3 and their families is not included in this review because usually state Departments of Education (i.e., the public schools) are not the lead agencies, there are different federal regulations, and the service context is quite different from school-based intervention. However, there are times that intervention practices overlap and are relevant for both early intervention and school-based programs. Last, we plan to draw content for the commentary from peer-reviewed journal articles although we will refer to landmark books or book chapters when relevant.

In this paper, we distinguish between intervention or instructional practices and comprehensive programs (Odom et al., 2010). We define practices as actions by teachers or other service providers, activities, and/or organizations of the environment to address a specific goal or outcomes (e.g., communicate in three-word sentences, social interaction with peers). Practices are more time-limited than programs in that they tend to be used until a specific learning goal is met. Comprehensive programs consist of a set of practices organized by a conceptual framework, cover multiple developmental or skill domains, are intensive (e.g., 20 + hours per week), and extend across a substantial period of time. In the commentary we will focus primarily on a discussion of practices.

## Historical Precedents to Education Interventions

Diagnosis of a condition such as autism implies that there should be a way of intervening to prevent, ameliorate, or improve the condition. The most well-known early programs for children with autism were based on different theoretical models. Following a psycho-analytic model, Bettelheim (1967) theorized that autism resulted from emotionally cold mothers and their failure to establish relationships with their children. His “treatment” involved removing the child from the toxic maternal influence (i.e., a parentectomy) through enrollment in a residential program, with many parents later reporting iatrogenic effects for their children. In 1968, Churchill, Ferster, and DeMyer (1971) convened a colloquium at Indiana University Medical School to report and reflect on the most current research of the time. In addition to a discussion about the need for reliable diagnostic criteria for autism (i.e., a foreshadowing of the DSM process), two researchers described theories and intervention practices that countered Bettelheim’s psychogenic approach. Ivar Lovaas (1971) described the remarkable success of his application of behavioral principles to teaching strategies for young children with autism, which laid the groundwork for the intensive behavior therapy movement described in the next section. The second pioneer at the Indiana Colloquium was Eric Schopler (Schopler & Reichler, 1971) whose research was actively involving parents in their children’s learning and development. He and colleagues also designed environmental accommodations that would make learning and independence in classrooms more feasible for autistic children. These techniques evolved into the TEACCH program that is used frequently in the U.S. and internationally. In most programs for children and youth with autism today, one can identify practices that can be directly traced back to these early pioneering approaches.

By the year 2000, knowledge about instructional and intervention practices for children with autism had accumulated, and the National Academy of Science convened a committee to review the then current research. They produced an oft-cited and influential report entitled *Educating Children with Autism* (National Research Council, 2001). The report identified and described a variety of comprehensive programs for children with autism, many of which were operating in lab or community school settings. Importantly, the committee also identified effective, empirically-based intervention practices that focused on key areas of development of children with autism (e.g., social, communication, etc.), and specified the importance of intervention intensity (e.g., provision of services at least 25 h per week). The push of evidence-based medicine (Sackett et al., 1996) and requirements of federal law, extended the focus on evidence-based practices as the basis for education intervention. This evidence-based practice movement is described in a subsequent section and is a primary theme of current intervention practice in education.

## Key Themes in Education for Autistic Children and Youth

As noted, the last 40 years has brought about significant changes in the types and quality of intervention practices available for use in educational programs for children and youth with autism. Key themes that reflect these change are related to adult-led and naturalistic forms of instruction, education in inclusive settings, forms and functions of communication, use of aversive strategies and positive behavior intervention and support, collateral mental health conditions, postschool outcomes, technology-assisted instruction and intervention, ineffective interventions, and search for evidence-based practices. In this section of the commentary, we will use these themes as an organizational framework for describing educational intervention approaches.

### Adult-led and Naturalistic Forms of Instruction

In the 1980s, educational interventions for autistic children and youth were heavily adult-directed and based on applied behavior analysis (ABA). Over the last 40 years, adult-directed instruction has remained a primary education strategy, although “naturalistic” approaches have also emerged.

#### Adult-Directed and Discrete Trial Training

Drawing from the foundational studies in ABA (Wolf et al., 1964) and the elaboration of his own ABA work, Lovaas (1987) developed a discrete trial teaching program (DTT). In DTT, adults provide an instructional antecedent (i.e., teacher instruction), students emit a target behavior, and adults provide a consequence (potential reinforcer or error correction procedure) on a predetermined schedule (i.e., known as a three-term contingency). Usually adults provide a set of trials (i.e., call massed trials) targeting a specific skill. In Lovaas’ program, autistic children received a minimum of 40 h per week and had significantly better outcomes than autistic children who had received less intensive interventions of ten hours per week (Lovaas, 1987). The positive effects of this adult-directed approach were confirmed in a 4 ½ year follow-up of children in the study (McEachin et al., 1993) as well as international replications of the approach when conducted in schools (Eikeseth et al., 2002). As the outcomes of the research by Lovaas and colleagues became more well-known, parents began to advocate for use of ABA by school personnel, including through litigation (Yell & Drasgow, 2000). The use of DTT has become pervasive as an educational strategy. Discrete trials have been and continue to be used to teach many skills addressed in educational settings (Hall, 1997). More recent research conducted in schools has evaluated performance feedback on educators’ delivery of instruction (McKenny & Bristol, 2015), the effectiveness of extending one-to-one DTT to group instruction (Taubman, et al., 2001), and a comparison of immediate to delayed reinforcement on skill acquisition (Carroll et al., 2016).

#### Naturalistic Intervention

In the 1980s, researchers began to realize that the use of highly structured, adult-led DTT sessions as the primary approach alone had shortcomings, such as the failure to generalize learned skills, prompt-dependence, escape/avoidance behaviors, and the lack of spontaneous responding (see Schreibman et al., 2015). An alternative to adult-led intervention, incidental teaching was first established by Hart and Risley in 1975 with young children from low income families and later modified by McGee (1983) for children with autism. The intervention was still based on ABA, but “naturalistic” in that the antecedent was a motivating context (i.e., often in a typical classroom routine or activity), involved child-initiation of the targeted behavior (i.e., rather than responding to adult-initiated instruction), and provided access to reinforcers natural to the environment (McGee et al., 1985). Pivotal Response Training (Koegel et al., 1987) was an early application of such a naturalistic behavioral approach and more recently has been adapted for use as a classroom-based program for early elementary-aged children with autism (Suhrheinrich et al., 2020).

In 2015, researchers using naturalistic behavioral intervention approaches that were conceptually situated in a developmental theoretical perspective determined that their approaches shared many common features. They classified their interventions under an umbrella term called Naturalistic Developmental Behavioral Interventions (NDBI, Schreibman et al., 2015). These interventions employed the naturalistic behavioral intervention factors noted previously and incorporated developmental science in identifying the focus and scope of learning outcomes. Preschool examples of these included Enhanced Milieu Teaching (EMT) (Kaiser & Hester, 1994), Joint Attention, Symbolic Play, Engagement, and Regulation (JASPER; Kasari et al., 2006) and its adaptation for school settings (Advancing Social-Communication and Play (ASAP; Boyd et al., 2018), and the classroom versions of the Early Start Denver Model (Vivanti et al., 2019) and Pivotal Response Teaching (Suhrheinrich, et al., 2020). Despite the large body of evidence demonstrating the efficacy of NDBIs (Tiede & Walton, 2019), additional research is still needed to understand how to adapt NDBIs for older children, such as those attending K-12 schools (Schreibman et al., 2020).

### Education in Inclusive Settings

In 2018, about 40% of autistic children who qualify for special education spend 80% or more of their time in general education (U. S. Department of Education, 2020) as compared to 9% in 1992–93 (U. S. Department of Education, 1995). One key influence is the broadened conceptualization of autism that identifies children and youth who do not have accompanying intellectual disability and who could benefit from the general education curriculum. With greater participation in general education has come a shift from a traditional life-skills intervention orientation to one that also includes a focus on academic and social skills (Spooner & Browder, 2015). A second key influence has been the Individuals with Disabilities Education Improvement Act (IDEA) (2004), which requires inclusion in the least restrict environment to the extent that an appropriate education can be provided in that context. Although a four-fold increase in inclusive placements have occurred over the 25 year reporting period just noted, it is also important to note that more than 60% of autistic children qualifying for special education are only partially included (i.e., less than 80% of the time) or are primarily in a self-contained special education class.

## Intervention and Instruction in Content Areas

In addition to the more general foci on adult-directed and naturalistic interventions and instruction in inclusive settings, a large proportion of the intervention research literature has focused on specific areas of learning needs for students with autism.

### Explicit Instruction for Academic Skills

A growing body of research demonstrates that a variety of intervention approaches may explicitly address academic and school related goals (Plavnick et al, 2015). For example, in their review of evidence-based practices, Steinbrenner et al. (2020) identified 25 practices that address academic and/or school readiness skills. Task analysis, direct instruction, response-prompting procedures (e.g., model-lead-test), visual supports (e.g., graphic organizers), modeling, and time delay are all practices that have been linked to growth in academic skills (Fleury et al., 2014).

Various intentional combinations of the above explicit instructional practices implemented in inclusive and self-contained settings have demonstrated the capacity to improve reading comprehension across core content areas (Knight & Sartini, 2015), writing (Asaro-Saddler, 2016), and math skills (King et al., 2016) for autistic students with and without intellectual disability. Furthermore, structured inquiry and explicit instruction of social studies content (Schenning et al., 2013) and science terms and their applications (Taylor et al., 2020) have promoted growth in these subject areas for students across developmental and functioning levels. Building these academic skills to each student’s potential creates a foundation that will support independent living and future educational and vocational choices.

### Interventions to Promote Social Engagement, Skills and Relationships

Difficulties engaging in social interactions with peers and adults, as well as establishing social relationships has always been one of the defining features of autism. Intervention approaches that have been used in education to promote social outcomes for children and youth with autism often employ adult-led and individualized skill training, peer-mediated approaches, and group-based social skills training.

#### Adult-led Teaching and Reinforcement Interventions

Some of the earliest intervention research on behavior of socially isolated children (i.e., autism was rarely used as a descriptor early on), employed adult use of reinforcement contingent on interaction with peers (Allen et al., 1964). From the early studies onward, investigators have used a variety of prompting, direct teaching techniques, and reinforcement with individual autistic children and youth, such as approaching peers and sharing desirable items like candy for young children (Kirby & Toler, 1970) and video games with adolescent peers (Gaylord-Ross et al., 1984), teaching social skills using scripts to support and prompt interaction on playgrounds (Krantz & McClannahan, 1993), and directly teaching play skills (Haring & Lovinger, 1987). Most recently, researchers have used a variety of other instructional approaches, such as self-management, video-modeling, and social narratives, to effectively promote autistic children and youth social engagement with peers (McKeithan, & Sabornie, 2020).

### Social Reciprocity and Peer-Mediated Interventions

In 1977, Strain and Shores published an influential paper noting that much of the previous social intervention research focused on promoting individual social behaviors rather than reciprocal social interactions, which is the more natural basis for social exchanges. In subsequent research, they developed a peer-mediated approach in which typically developing children socially engaged children/youth with autism in ways that led to reciprocal interactions (Strain, et al., 1979). The early peer-mediated intervention research did increase the reciprocal social interaction of autistic children (Odom & Strain, 1986) and more recently, Kasari et al. (2012) reported positive changes in peer social networks when a peer-mediated intervention approach was employed in schools. To extend the peer-mediated approach to support autistic children’s/youth’s social relationships, Haring and Breen (1992) designed a social network intervention in which social groups of peers supported social engagement in multiple activities during a school day. Both peer-mediated and peer-social network intervention have been adapted and employed in elementary (Kamps et al., 2015) and high schools (Carter et al., 2019), with this research literature remaining currently quite active (McKeithan & Sabornie, 2020).

#### Group-Based Social skills Training

For students with autism who are able to participate in psycho-educational group instruction, social skills training group interventions have been developed, more often for middle and high school age groups. Generally, the intervention is led by an educator, has a sequenced set of lessons, teaches specific skills (e.g., emotional recognition in others, problems solving in social situations), and may have homework assignments. Although these interventions have often been delivered in clinical setting (Jonsson et al., 2019), there are examples of social skills training programs, such as the Program for the Education and Enrichment of Relational Skills (PEERS®) (Laugeson et al., 2014), that have been employed in school settings, generating positive outcomes in terms of skill acquisition for participants with autism.

### Forms and Functions of Communication for Autistic Children and Youth

Communication is an essential developmental skill with vast variation in patterns of acquisition and outcomes for students with autism (Tager-Flusberg et al., 2005). Communication interventions have evolved in the past 40 years from focusing mostly on oral “expressive” language outcomes to thinking in tandem about communicative form (e.g., sign, AAC) and function across pragmatic contexts, as well as building foundational skills (e.g., joint attention).

#### Communication and Verbal Language Skills

Interventions targeting verbal communication skills for students with autism have fallen into three categories: behavioral, developmental, and naturalistic (Sandbank et al., 2020). The earliest intervention approaches used ABA principles and discrete trial training, generating positive effects on expressive language in students with ASD (e.g., Reichow et al., 2018). An individual adult-led therapy approach, sometimes called a pull-out model and usually not as structured as DTT, continues to be a primary mode of speech therapy in schools. A variety of other approaches that include behavioral practices (e.g., antecedent-based interventions, video-modeling, functional communication training) have been shown to produce positive communication outcomes (Steinbrenner et al., 2020). Developmentally based approaches, such as SCERTS (Social-Communication, Emotional Regulation & Transactional Supports) (Prizant et al., 2003), have been applied with autistic students to address verbal communication outcomes. Although originally designed as a family based-model, Morgan et al. (2018) has documented the efficacy of SCERTS in schools. In general, developmental interventions have shown some positive effects on foundational social communication outcomes for students with autism but with little evidence of cascading effects on language (e.g., Sandbank et al., 2020).

An alternative approach to pull-out intervention, sometimes described as a “push-in”, is naturalistic in nature and focuses on the social and functional use of language in context. For young autistic children, NDBIs (see previous discussion) are often used to enhance language acquisition. Though widely supported by randomized controlled trials (Sandbank et al., 2020), these are more often employed in clinic or home settings rather than classrooms, with some exceptions [e.g., classroom applications of EMT (Dubin et al., 2019)]. In current practice, service providers employ an individualized, eclectic blend of strategies from these behavioral and naturalistic approaches to target verbal communication skills.

#### Interventions for Nonverbal Children and Alternative and Augmentative Communication (AAC)

AAC is an alternative to teaching oral communication for autistic children and youth who are nonverbal (Iacono et al., 2016). Notably, there is also consistent evidence that AAC does not inhibit and often supports the development of spoken language for many autistic children with limited verbal communication skills (Schlosser & Wendt, 2008). AAC offers opportunities to build communication skills to augment limited spoken language, including no- and low-tech AAC (e.g., sign language), as well as high-tech AAC. Over the past 40 years, the proliferation of technology has changed options for AAC, especially high-tech AAC such as speech-generating devices (SGDs), which now include apps that can be used on tablets replacing dedicated SGDs (Lorah et al., 2015).

High-tech SGDs can be programmed for more diversity of vocabulary and communicative functions and have improved the communication of children with autism (Logan et al., 2017). However, it is critical to recognize that the SGDs provide an alternative mode of communication but may need to be paired with strategies to enhance communication use in typical contexts. Notably, recent work in schools has combined AAC more intentionally with other interventions such as peer-mediated intervention (e.g., Thieman-Bourque et al., 2017) and JASPER (e.g., Kasari et al., 2014) to increase communication of children with autism. Low-tech AAC has also proven effective, such as the Picture Exchange Communication System (PECS; Frost & Bondy, 2002). PECS uses ABA strategies combined with a concrete representation of physically exchanging symbols to support understanding of early communicative exchanges and contributes to positive gains in communication (Ganz et al., 2012).

## Aversive Strategies and Increased Focus on Positive Behavior Supports and Intervention

Although challenging behavior is not a defining characteristic of ASD, limited social communication skills and the strong adherence to ritual and routine can lead to challenging behaviors for some autistic children and youth (Esteves et al., 2021). The effectiveness of ABA to reduce challenging behavior has been demonstrated through research spanning the previous 40 years (Matson, et al., 1996), with some of those interventions employing aversive consequences (e.g., water spray, lemon juice, mild shock) for extreme and dangerous behavior (Gerhardt et al., 1991). In the 1980s, concerns were raised about the confirmed misuse of aversive procedures with recommendations to disallow their use (Berkman & Meyer, 1988). Heated debates ensued in which some advocates proposed that individuals should have the right to effective treatment (which could include aversives) for serious challenging behavior such as self-injury (Van Houten et al., 1988). Opponents questioned the ethics of using aversives when alternative nonaversive intervention strategies were available (Horner, et al., 1990). A major contribution to current policies was the seminal work of Iwata et al. (1994), who demonstrated that challenging behaviors could serve multiple functions. This research paved the way for use of functional behavior assessment prior to designing any intervention plan (Horner et al., 2002).

The national Positive Behavioral Interventions and Supports (PBIS) movement arose out of the heated debates of the 1980s (Dunlap et al., 2011). The technology of PBIS included behavioral assessments that could identify the function the problem behavior, selection of strategies that would prevent a behavior from occurring if possible, and teaching alternative and adaptive behaviors rather than focusing solely on suppressing maladaptive ones. Data-based decision making, developing functional skill sets, and being respectful of one’s dignity serves as the guiding principles of PBIS today (Kincaid, 2018).

This positive approach to supporting individuals who displayed challenging behavior was appealing to many families and agencies, including the federal government. In the 1997 and 2004 reauthorizations to the IDEA, the U.S. Department of Education mandated that, when students display challenging behavior that interferes with learning school personnel must conduct a functional behavior assessment before developing a behavior intervention plan and consider positive behavior interventions to support the student. Although the possibility of using aversive intervention still exists when all other alternatives have been tried, the mandate changed the way schools responded to challenging behavior. Currently, many students with autism attend schools that employ School-wide Positive Behavioral Interventions and Supports (SWPBIS). This program focuses on a tiered set of interventions that begins with proactive, preventative strategies and moves to progressive more intensive behavioral interventions as needed. SWPBIS has been employed in more than 26,000 school across the United States (Sugai & Horner, 2020).

### Collateral Mental Health Conditions

Across the decades, behavioral and educational intervention providers have taken a broader view of the factors associated with behaviors viewed as problematic (e.g., stereotypies, self-injurious behavior, tantrums and meltdowns), and there has become a greater awareness of mental health conditions as one of the possible underlying factors. In the DSM-5, the diagnosis of ASD includes the specification of association with another mental or behavioral factor when applicable (APA, 2013). Mental health conditions are highly prevalent in individuals with ASD and rates are higher than those in the general population. The highest reported conditions include anxiety, depression, ADHD, schizophrenia, sleep–wake disorders, conduct disorders, bipolar disorders, and obsessive–compulsive disorders (Lai et al., 2019). Although few school-based interventions target mental health, there are some exceptions. Cognitive behavioral/instructional strategies, including cognitive behavioral therapy, are identified as an evidence-based practice for mental health outcomes (Steinbrenner, 2020). While most evidence of cognitive behavioral therapy for anxiety has been clinic-based, recently studies have adapted interventions for the school settings and found positive effects. For example, Luxford et al. (2017) used the *Exploring Feelings CBT to Manage Anxiety* curriculum to teach students to understand and manage their emotions. In addition, a family- focused intervention, *Facing Your Fears*, has also been adapted for the school setting and incorporates parent educational sessions (Reaven et al., 2020).

### Post-School Outcomes

The period of secondary transition for youth with autism and other developmental disabilities has changed significantly over the past 40 years. Although post-school outcomes are still less than optimal for this population, gains have been made. In the 1980s and early 1990s outcomes for young adults with autism were restrictive in nature, with most living at home and working in sheltered workshops or attending day activity programs. Today, postschool outcome data from the National Longitudinal Transition Study 2 (NLTS2) indicate that 21% of young adults with autism are employed full time in paid work in the community, with nearly 36% having attended a 2- or 4-year college after exiting high-school (Roux et al., 2015). Conceptual models of post-school transition have evolved over time, which has advanced the field. In the 1980s, early models of transition were uni-dimensional equating transition success with competitive employment (e.g., Will, 1984). In later years transition success was expanded to encompass community adjustment, which included not only a pillar of employment, but also pillars of community living and social networks (Halpern, 1985). Federal laws have also paved the way for increased resources and regulations regarding transition programming for youth with autism. The 1990 reauthorization of IDEA with its mandate of individual transition plans for youth with disabilities beginning at age 16, was instrumental in prescribing specific elements that must be incorporated in an Individualized Education Program, including specific post-school outcomes and needed transition services. These targets are critical and serve to guide the programming and curriculum at the secondary level.

The early work of Kohler (1996) provided a general Taxonomy for Transition Programming that guided subsequent identification of educational transition practices for secondary students with autism by the National Technical Assistance Center on Transition (NTACT, 2020). Outcome data for young adults with autism and other developmental disabilities have indicated that more time spent in general education classrooms is associated with increased academic skills and knowledge, receipt of a high school diploma, and/or increased access to typical peers, which promotes social relationships and inclusion and ultimately more integration in the community (Landmark et al., 2010). Youth participation in both paid and unpaid work experiences during high school is also correlated with better outcomes in adulthood, including competitive or supported employment, number of hours worked, and hourly wage (Test et al., 2009). Family involvement in the educational and transition planning process is also critical, particularly given that family members are often the lifelong caregivers or support providers. One example of a research based program that establishes a collaborative planning process involving families and teachers in transition planning is the COMPASS model developed by Ruble et al. (2018). Although such planning and preparing for the post-school future is critical, many autistic students exit the school system with no support systems in place. Data published in the *National Autism Indicators Report: Transition Into Young Adulthood* indicates that 1 in 4 young adults with autism had no access to services since leaving high school (Roux et al., 2015).

Social skills and functional life skills, as well as vocational skills, are critical for supporting success in adulthood (Dell’Armo & Tasse, 2019). Intervention practices to address these skills have demonstrated efficacy for adolescents with autism. These include antecedent-based interventions, modeling, peer-based interventions, prompting, reinforcement, social skills training, task analysis, time delay, video modeling, and visual supports (Steinbrenner et al., 2020). However, comprehensive, empirically supported educational and employment programs for transition age youth with autism are limited. One exception is Project SEARCH. Project SEARCH is an intensive year long program for high-school students with disabilities that involves hands-on work experience along with skills training and placement assistance (Rutkowskia et al, 2006). The model integrates classroom instruction with on-the-job training and connects services and supports from educational and rehabilitation professionals to the employment setting. Wehman and colleagues (2020) have applied the Project SEARCH model with autistic adolescents and, in a series of studies culminating in a multisite randomized trial, have found consistently positive vocational outcomes and post-school vocational placements.

#### Self-Determination

For young adults with autism, self-determination is associated with positive post-school outcomes (Zalewska et al., 2016). Self-determination refers to the ability of, and opportunity for, students to make their own decisions and advocate for themselves (Shogren et al., 2015). Secondary students with ASD must be able to identify the types of supports and accommodations they need and must be able to articulate these needs to receive those services in college or on the job. However, due to the unique communication and social needs of students with ASD, specific educational and environmental components may be necessary to foster self-determined behavior (Wehmeyer et al., 2010). While no intervention approaches have included high school students with ASD exclusively, one evidence-based transition curriculum that has included a subpopulation of students with ASD and co-occurring intellectual disability is Self-Determined Learning Model of Instruction (SDLMI; Shogren et al., 2019). The SLDMI consists of three phases: (1) to define and set goals; (2) develop a self-management action plan and; (3) self-monitor and self-evaluation. SLDMI is associated with positive outcomes including increased self-determination, student-directed transition planning, increased access to the general curriculum, improved classroom behavior, and student attaining educationally relevant goals (Hagiwara et al., 2017).

### Technology-Assisted Instruction and Interventions

Advances in technology are ubiquitous in nearly everyone’s life today, and in fact space constraints allow only a brief coverage of this rapidly expanding intervention area. For this paper, we define technology-assisted interventions as “an electronic item/equipment, application, or virtual network that is used to intentionally increase, maintain, and/or improve daily living, work/productivity, and recreation/leisure capabilities of adolescents with autism spectrum disorders” (Odom et al., 2015, p. 3806). Forty years ago, the application of technology to instruction for individuals with disabilities was just emerging, with most of the applications bound to mainframe or then a new technology, desktop computers (Strain & Odom, 1985). Advances in smart-phones, tablets, telecommunication, virtual reality, artificial intelligence, and social media, all have had implications for use in education for students with autism. For example, tablets have been used as personal organizers that contain visual schedules, auditory prompts, and notetaking (Chelkowski et al., 2019). Smartphones and digital cameras also allow teachers to easily capture video examples of the skills that autistic children/youth learning and using those example as video-models (Dueñas et al., 2019). Technology has been used to deliver the content of instruction through traditional computer-assisted instruction ( LeBlanc et al., 2017), and tablets (Spooner et al., 2014). At this writing, we are in the midst of a pandemic and in-school instruction in many districts has halted. Teaching remotely through telecommunication and computer technology has become the modal form of instruction. Although not without its downsides (e.g., lack of access to the technology, knowledge of computer “etiquette”, absence of direct adult and peer mediation), there are empirical demonstrations of remote instruction for and learning by students with autism (Parsons et al., 2019). In addition, professional development of teacher and other service providers working with children/youth with autism has been delivered through didactic webinars, remote coaching, and self-paced instructional modules introducing EBPs (Sam et al., 2020).

## Ineffective Interventions

For children and youth with autism, advocates and purveyors have proposed interventions and treatment that at the least are not effective and at the most are harmful. From the field of health, interventions such as hyperbaric chambers and chelation therapy have been proposed as “cutting edge” therapies (Siri & Lyons, 2014), despite their limited evidence of efficacy and possible harm. The field of education is no different, and at times practitioners adopt ineffective or even harmful practices. The most prevalent in education has been the use of facilitated communication (FC). Originally developed in Australia in the 1970s, FC became popular in the United States in the 1980s (Biklen & Schubert, 1991). In this technique a facilitator (adult teacher or parent) supports (physically) an autistic individuals’ arm or hand as they spell out words that communicate their thoughts. Over the last 25 years more than 19 rigorous experimental studies have demonstrated that the origin of the communication is the facilitator, not the person with a disability (Ganz et al., 2017), and it is primarily viewed as a discredited instructional method. However, adapted forms of the FC approach have emerged recently under titles such as rapid prompting method and supported typing (Pena, 2019), with rigorous systematic reviews again finding no evidence of effects (Schlosser et al., 2019).

Other ineffective intervention approaches for children and youth with ASD that appear in practice also have been largely discounted. For example, auditory integration therapy is a technique that could be provided by a trained audiologist as part of a related service but has been discredited by the American Academy of Pediatrics (2010). Children/youth with autism often experience sensory issues (e.g., loud noises are disturbing, touch is aversive). In schools often “sensory” interventions are provided. There is evidence that the Sensory Integration Therapy™ developed by Jean Ayres (2005) can be effective if provided by a trained therapist (Schaaf et al., 2014). Conversely, a variety of other “sensory” interventions such as weighted vests, sensory diets, or sensory rooms have little evidence of effectiveness (Case-Smith et al., 2015), although they are often used in school. One approach to reducing the number of ineffective, sometime fraudulent, interventions is to identify and broadly disseminate information about practices that are effective.

## Evidence-Based Practices for Children and Youth with Autism

To counter the proliferation and use of ineffective and even harmful practices, there have been efforts to systematically identify intervention practices and programs that do have evidence of effectiveness. This evidence-based practice movement in education took the lead from the evidence-based medicine movement, originating with Cochrane (1972) and carried forward by Sackett et al. (1996), as well as several organizations that conduct and post systematic review of practices (e.g., Cochrane Collaboration, Campbell Collaboration, What Works Clearinghouse). Several research groups have conducted large scale, systematic reviews to identify studies that meet methodological standards and indicate positive effects on outcomes for autistic students. For example, the National Clearinghouse on Autism Evidence and Practice, reviewed the intervention literature from 1990 to 2017 and identified 28 EBPs for individuals with autism ages 0–21 (Hume et al., 2021; Steinbrenner et al., 2020). In an independent and parallel review, the National Standards Project reviewed the literature from 1957–2012, which identified 14 established practices for individuals under age 22 (NSP, National Autism Center, 2015). Comparison of an earlier review by NCAEP researchers (Wong et al., 2015) and the NSP review found substantial overlap between the two sets of practices (https://autismpdc.fpg.unc.edu/sites/autismpdc.fpg.unc.edu/files/imce/documents/Matrix%20NPDC%20NSP%20v3.pdf). For older youth with autism, the National Technical Assistance Center on Transition identified 24 EBPs related to transition outcomes for students across disability areas (NTACT, 2020). As the autism intervention literature continues to accelerate rapidly, the ongoing identification, dissemination, and most importantly, support for broad implementation of EBPs, will remain a critical need in the field.

## Schools as Contexts for Implementing Interventions

The research-to-practice gap is a challenge for school-based programs, despite the evidence of practices that generate positive outcomes for autistic children and youth as described previously. In this section, we describe features of the school context that pose challenges for implementation of effective practices. A variety of approaches, which we call the iSciences, have become prominent as methods to address these barriers to implementation. These approaches will be identified and described.

### Complexity of School Settings

Although the characteristics and settings for special education for children with autism have changed over the past 40 years, gaps between research and practice exists. Challenges to implementation of evidence-based practices relate to school structure, characteristics and preparation of personnel, and disparities in education settings and services.

#### Structure of Schools

Serving autistic students in school settings is a complex undertaking, as the population is notably heterogeneous, which requires individualized programming across a variety of settings and professionals. Selecting practices that are effective across context and feasible for implementation by general educators, special educators, paraprofessionals, and related service providers is difficult (Barry et al., 2020), as is coordinating opportunities for thoughtful collaboration and professional development for the team (Sinai-Gavrilov et al., 2019). The competing demands for school resources continue to mount, with increased pressure on schools to have students perform well on high stakes testing and meet rigorous academic requirements for graduation. This pressure limits autistic students’ access to specialized instruction related to social interaction and communication, transition preparedness, life skills, and coping skills (Snell-Rood et al., 2020). These structural factors may affect overall program quality for students with autism, which has proven to be low in schools across the country (Kraemer et al., 2020). High quality programs serve as a foundation for the implementation of EBPs, and thus poor program quality, as measured by school and classroom climate and instructional features, may itself serve as barrier to EBP implementation (Odom et al., 2018).

#### Personnel

Effective education for all students with ASD requires knowledgeable and skilled school personnel. Survey results reveal that pre-service preparation programs may not sufficiently prepare teachers to use evidence-based practices (Morrier et al., 2011), or prepare principals to arrange effective inclusion experiences (Lyons, 2016). Even if program graduates are well prepared, students with autism will not benefit from their skills if educators leave the field. The U.S. Department of Education consistently identifies special education as an area of teacher shortage (Cross, 2017). In their review of the attrition and retention literature, Billingsley and Bettini (2019) found that special educators are more likely to leave as a result of factors such as demanding working conditions, and a lack of support from administrators, colleagues, and paraprofessionals.

Creating systematic professional development (PD) opportunities are likely to limit attrition, support school personnel that did not receive autism focused pre-service training and provide updated information on research based practices. An essential feature of an effective PD system is the use of competent coaches, identified in the implementation science literature as a critical driver for sustained change (Fixsen et al., 2009). Coaching has shown to be far more effective in changing teacher practices than traditional PD (e.g., workshop training alone). Paraprofessionals also are in need of PD, particularly in the use of evidence-based practices (Barrio & Hollingshead, 2017). Ideally the PD would provide opportunities for fostering interdisciplinary collaboration and the identified benefits for the students they serve (Biggs, 2016).

#### Disparities in Schools

Another challenge plaguing schools is the issue of disparity, which includes disparities at district, school, and child levels. At school and district levels, there are differences in resources such as funding and personnel (e.g., Mason-Williams, 2015), which may impact the capacity of schools to uptake and sustain evidence-based approaches for serving children with autism. At the child level, children from minoritized racial and ethnic groups, as well as children of parents with lower levels of education are less likely to receive related services or autism-specific therapies than their peers (Smith et al., 2020), and there are also diagnostic disparities (Maenner et al., 2020). Collectively, these disparities may be contributing to racial/ethnic and socioeconomic inequalities throughout the education experience for students with autism as well as during transition out of schools (Eilenberg et al.; 2019) and beyond.

## Emergence of iSciences

To address the research to practice gap in educational programs for autistic children, and the barriers described previously, the iSciences have emerged. Although all the approaches we describe here do not all begin with the letter “I”, when employed in education they have in common the goal of moving evidence-based practices about effective instruction and intervention into use in educational programs for children and youth with autism. Space constraints prevent all but an acknowledgement of their foci. *Information science* is the “effective collection, storage, retrieval, and use of information (p. 2570, Saracevic, 2009) and has been an integral part of efforts to share information about evidence-based practices (Sam et al., 2020) and support data-based decision-making. *Improvement science* (Lewis, 2015) has emerged as an iterative process for developing and testing interventions in schools as well as improving the applications of interventions within specific school contexts. Similarly, *Diffusion of Innovation theory* (Rogers, 2003) attempts to explain how a new practice or idea spreads through social systems, and Dingfelder and Mandell (2011) have proposed that it may be an effective approach for promoting the use of effective practices. *Dissemination theory*, an offshoot of Diffusion Theory (Dearing, 2008), focuses more on societal “sectors” as social networks and implementation in complex organizations, which is also a feature of implementation science. Last, *Implementation science*, in its application for school-based programs, is the process for promoting the practitioner’s use of a program or intervention by addressing factors in the context (e.g., professional development, coaching) or in the system of influence operating outside of the immediate context (e.g., administrative leadership and support) (Odom et al., 2020). Although all of these iSciences hold promise in closing the research to practice gap, to date Implementation Science has been employed most often in educational programs for students with autism.

## Conclusion

Our understanding of autism and how to best serve autistic students in school settings has changed considerably since the 1980s, a time period now described by autistic adults as “The Lost Generation,” due to their limited access to early diagnosis, intervention, and evidence-based practices (Lai & Baron-Cohen, 2015). Across the 40-year history, since the publication of DSM III, educational interventions and services for children and youth with autism have expanded greatly. The evolution of the DSM system, as well as reliable and valid diagnostic instruments (Lord et al., 2012) has created a more reliable process for identifying autism in educational systems (i.e., in the U.S. educational diagnoses are very similar to the DSM criteria). This, in turn, has led to greater awareness of the need for interventions and instruction that can be delivered in school contexts. In addition, as educational and behavioral theory expand and evolve, we see shifts in how and where students with autism are served, the immediate and post-school outcomes we value, and the methods we use to reach those outcomes. These changes contribute to the need for new knowledge, and thus our rapidly accelerating intervention literature base. The more recent advances in and adoption of strategies that support the use of EBPs in school systems increase the likelihood of closing the research to practice gap that continues to exist in education and uphold the promise of a free and appropriate education for students with autism.

## Footnotes

1 In this commentary we will use the terms “autism” and “autism spectrum disorder (ASD)” synonymously as well as both person-first and identify-first descriptors.
